# Identification of key modules and genes associated with breast cancer prognosis using WGCNA and ceRNA network analysis

**DOI:** 10.18632/aging.202285

**Published:** 2020-12-09

**Authors:** Xin Yin, Pei Wang, Tianshu Yang, Gen Li, Xu Teng, Wei Huang, Hefen Yu

**Affiliations:** 1 Beijing Key Laboratory of Cancer Invasion and Metastasis Research, Department of Biochemistry and Molecular Biology, School of Basic Medical Sciences, Capital Medical University, Beijing 100069, China

**Keywords:** breast cancer, differential expression analysis, WGCNA, lncRNA, PPI network

## Abstract

Breast cancer is one of the leading causes of cancer-associated mortality in women worldwide and has become a major public health problem. Although the definitive cause of breast cancer is not known, many genes sensitive to breast cancer have been detected using advanced technologies. Our study identified 3301 differentially expressed lncRNAs and mRNAs between tumor and normal samples from The Cancer Genome Atlas database. Based on the gene expression analysis and clinical traits as well as weighted gene co-expression network analysis, the co-expression Brown module was found to be key for breast cancer prognosis. A total of 453 genes in the Brown module were used for functional enrichment, protein-protein interaction analysis, lncRNA-miRNA-mRNA ceRNA network, and lncRNA-RNA binding protein-mRNA network construction. *GRM4*, *SSTR2*, *PARD6B*, *PRR15*, *COX6C*, and lncRNA *DSCAM-AS1* were the hub genes according to protein-protein interaction, lncRNA-miRNA-mRNA and lncRNA-RNA binding protein-mRNA network. Their high expression was found to be correlated with breast cancer development, according to multiple databases. In conclusion, this study provides a framework of the co-expression gene modules of breast cancer and identifies several important biomarkers in breast cancer development and prognosis.

## INTRODUCTION

Breast cancer (BRCA), a neoplasm of the epithelium, is one of the most common cancers worldwide and the most common cause of cancer death among women, with more than 42,000 cases reported in 2020 [1]. Primary BRCA itself is not fatal; however, BRCA cells metastasize easily to other organs, including the brain, lung, liver, and bone, which is the main cause of BRCA-related death [2]. Despite advances in diagnosis and treatment strategies, nearly 60% of BRCA cases are diagnosed at advanced stages when chances of mortality are very high [3]. Between 25% and 50% of patients diagnosed with BRCA will eventually develop into deadly metastases, often decades after the diagnosis and removal of the primary tumor [4]. While significant progress has been made in understanding the progression and prognosis of BRCA, the specific mechanism underlying BRCA progression remains unclear. Currently, clinicopathological factors, such as tumor size, axillary lymph-node status, and pathologic et al. have been used most frequently to predict diagnosis and prognosis of breast cancer patients [5]; nevertheless, their use alone was insufficient for choosing therapeutic strategy and predicting BRCA prognosis. BRCA is multifactorial disease most caused by genetic mutation [6]. Numerous genetic alternations influence BRCA progression and indicate BRCA prognosis. It is meaningful to find biomarkers to evaluate BRCA progression and prognosis, which might prompt patients to take therapy at the early stage and improve their survival rate. Thus, further research is needed to diagnose and treat BRCA more effectively and thereby improve the survival and prognosis of BRCA patients.

Long noncoding RNAs (lncRNAs) are endogenous cellular RNAs that have recently been detected in various cancers, including BRCA. They are involved in multiple biological processes and are promising candidates for the diagnosis, prognosis, and treatment of BRCA [7, 8]. The lncRNA transcripts are greater than 200 nt in length without the ability to encode proteins [9]. lncRNAs can hybridize to the overlapping sense transcript and modulate alternative splicing patterns or generate endo-siRNAs as well as bind with specific protein partners to modulate protein activity, alter protein localization, or serve as a structural component that allows formation of larger RNA–protein complexes [10]. Analogical to protein-coding genes and miRNAs, lncRNAs can participate in cancer progression. Several lncRNAs have been found to be aberrantly expressed in BRCA. For example, lncRNA *HOTAIR* is activated by carcinoma-associated fibroblasts via TGF-β1 secretion to promote BRCA metastasis [11]. Similarly, lncRNA *H19* is a sponge for miR-200b/c and let-7b that induces the expression of miRNA targets *Git2* and *Cyth3* to promote cell migration [12].

Recent technological advances, including microarray and high-throughput sequencing, have improved our understanding of molecular biology, particularly with regard to lncRNAs. The weighted gene co-expression network analysis (WGCNA) algorithm is a novel biological approach used to identify highly correlated gene modules and key genes based on gene expression data [13, 14]. WGCNA simplifies the interpretation of thousands of genes and constructs a co-expression network on the basis of similarities in expression profiles among samples [15]. Highly co-expressed and closely connected genes are enriched in the same module, which is conserved across phylogenies and enriched in protein-protein interactions (PPIs) [16]. WGCNA addresses one drawback of traditional microarray analysis, which only focuses on individual genes and ignores the correlations between genes [17, 18]. By constructing gene networks between normal and tumor tissue expression data and combining them with clinical traits, it is possible to identify potential biomarkers or therapeutic targets [19]. For example, WGCNA was used to identify eight lncRNAs that significantly reduced overall survival in laryngeal cancer, which may be important biomarkers for laryngeal cancer development and disease progression [20]. In one prognostic study performed using WGCNA, the lncRNA *TRPM2* was found to be a competing endogenous RNA (ceRNA) that promotes the proliferation and inhibits the apoptosis of BRCA cells via the TRPM2-AS/miR-140-3p/PYCR1 axis [21].

In the present study, we applied WGCNA to BRCA gene expression data (including lncRNA and mRNA expression data) from The Cancer Genome Atlas (TCGA) database to identify key co-expression modules in BRCA patients compared to healthy controls. These modules were closely related to clinical traits in patients with BRCA. Genes in the identified modules may affect the development of BRCA. The co-expression Brown module was selected for further analysis because it was significantly associated with prognosis of BRCA. In addition, analysis of the lncRNA-miRNA-mRNA and lncRNA-RNA binding protein (RBP)-mRNA networks may offer novel insights into the molecular mechanisms of BRCA and provide novel techniques for the diagnosis of BRCA to improve BRCA prognosis.

## RESULTS

### Identification of differentially expressed lncRNAs and mRNAs and gene function enrichment analysis of DEmRNAs

**Figure 1:**
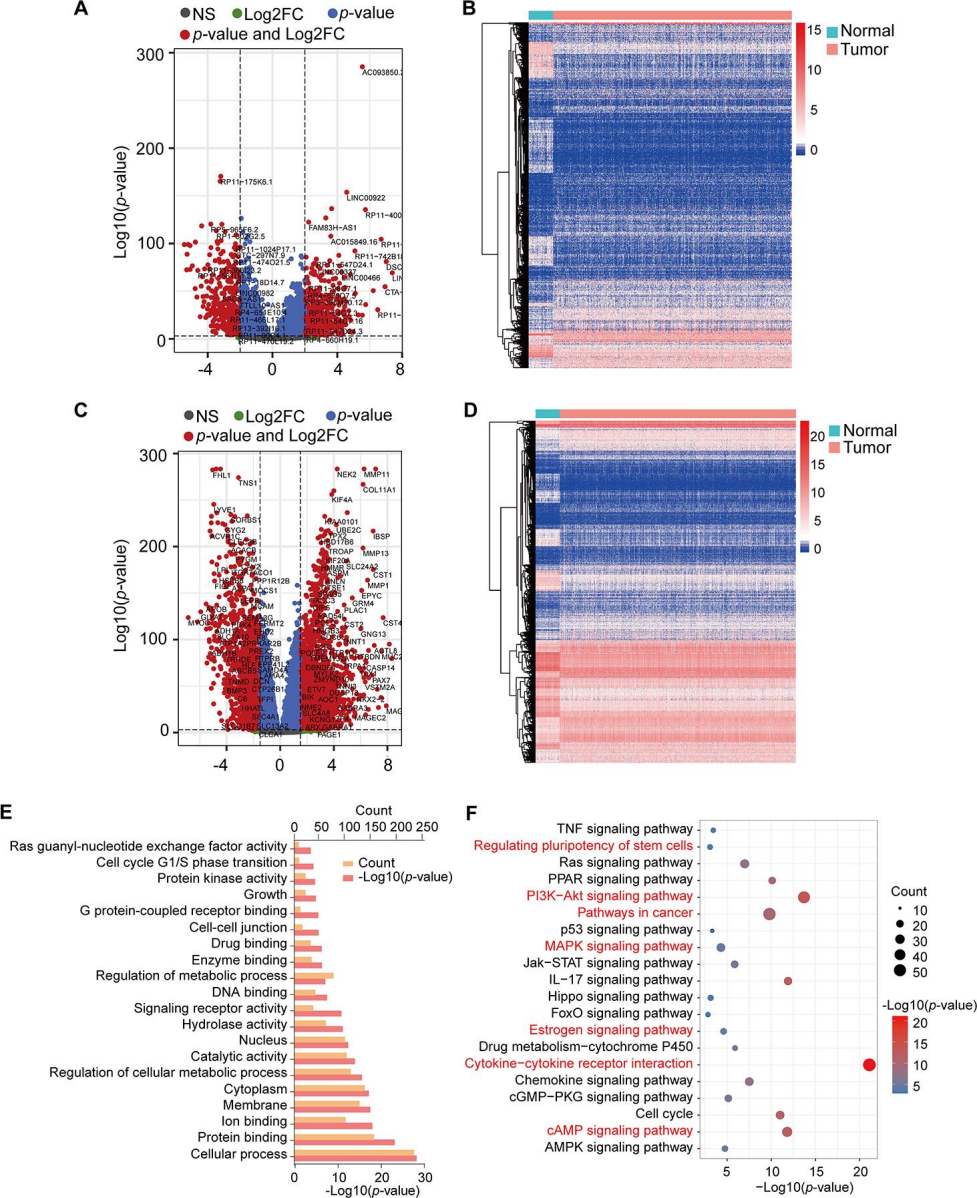
**Volcano plots, heatmap, and gene enrichment analysis of DElncRNAs and DEmRNAs.** (**A**) Volcano plot of DElncRNAs. (**B**) Heatmap of DElncRNAs. (**C**) Volcano plot of DEmRNAs. (**D**) Heatmap of DEmRNAs. NS: no significant, Log2FC: |Log2FC|>2, *p*-value: *p*-value<1e-3, *p*-value and Log2FC: *p*-value<1e-3 and |Log2FC|>2. (**E**) GO enrichment of DEmRNAs. (**F**) KEGG pathway enrichment of DEmRNAs. Red pathways are common with DEmRNAs of the Brown module.

The TCGA RNA-seq expression dataset contains lncRNAs, mRNAs, and miRNAs obtained from 1098 BRCA patients and 113 healthy subjects. After the raw data were normalized, 3301 differential genes were identified between the tumor and normal samples using |log2FC| > 2 and adj-*p* < 1e-3 in the DESeq2 R package. In total, 2008 and 1293 genes were increased and decreased in tumor samples, respectively (Supplementary Table 1). Volcano plots and heatmaps were plotted to show the distribution of 853 lncRNAs (DElncRNAs) (Figure 1A, 1B) and 2448 mRNAs (DEmRNAs) (Figure 1C, 1D) that were differentially expressed in BRCA in comparison to the normal samples.

We obtained the target genes of DElncRNA using the RAID 2.0 database and identified 237 target genes, which had 40 overlapping genes with DEmRNAs (Supplementary Table 2). The KOBAS online database was used to conduct GO and KEGG pathway annotation analyses for the 2645 mRNAs (DEmRNAs and target genes). Enrichment results were visualized by the R package ggplot2 (Figure 1E, 1F). GO analysis showed that these genes were significantly enriched in protein binding, signaling receptor activity, enzyme binding, and G protein-coupled receptor binding (Figure 1E). Moreover, cytokine-cytokine receptor interaction, PI3K-Akt signaling pathway, IL-17 signaling pathway, cAMP signaling pathway, cell cycle, PPAR signaling pathway, and Ras signaling pathway, among others, were also obtained from KEGG pathway enrichment analysis (Figure 1F, Supplementary Table 3).

### Construction of co-expression modules of BRCA by WGCNA

**Figure 2:**
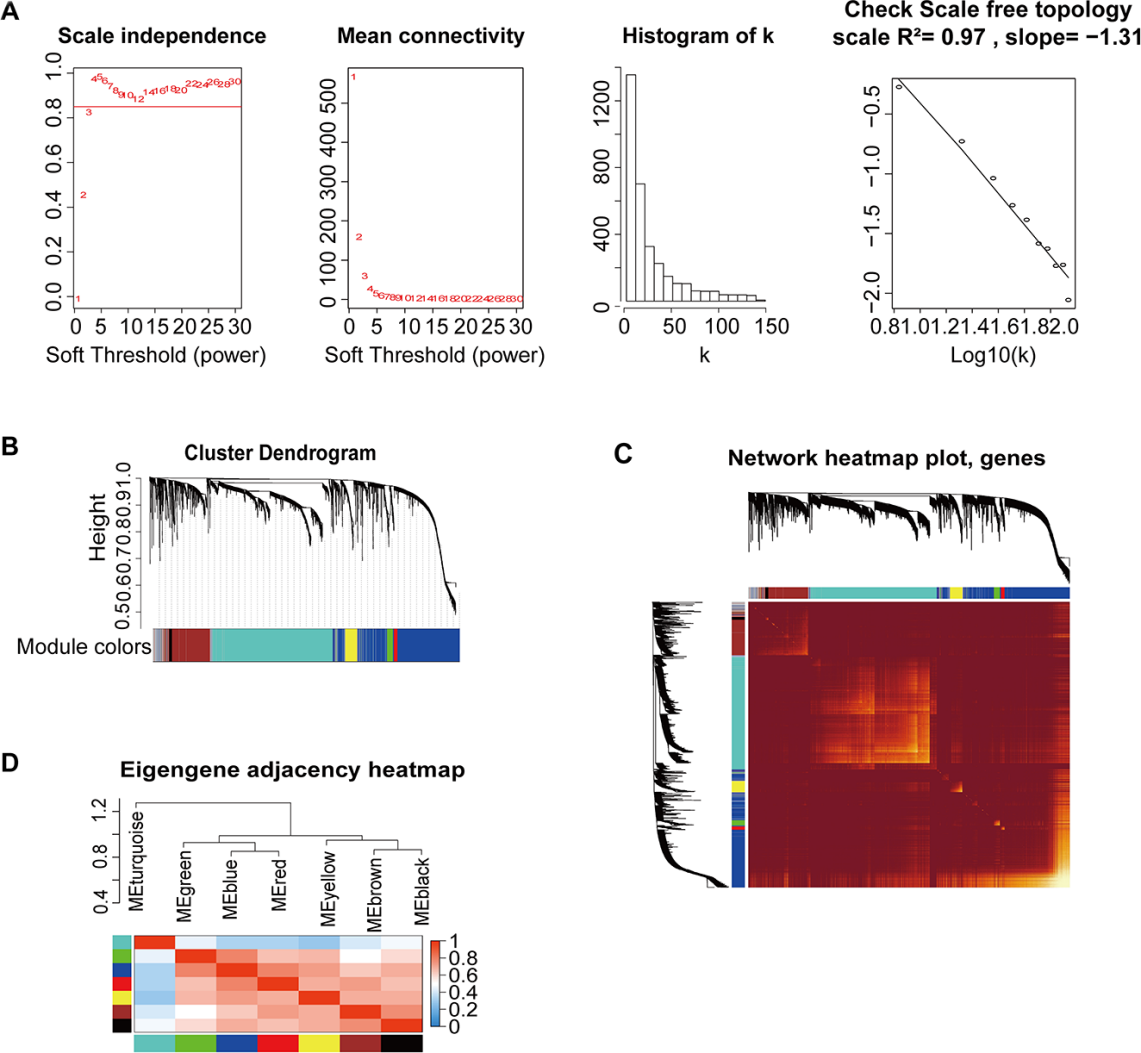
**Construction of co-expression modules based on BRCA RNA-seq data from TCGA database by WGCNA.** (**A**) Analysis of network topology for various soft-threshold powers. Check scale-free topology; the adjacency matrix was defined using soft-thresholds with β=4. (**B**) Clustering dendrograms of genes, with dissimilarity based on topological overlap, together with assigned module colors. (**C**) Heatmap depicting the topological overlap matrix (TOM) among genes based on co-expression modules. A redder background indicates a higher module correlation. (**D**) Visualization of the gene network using a heatmap plot.

All DEmRNAs, including 853 DElncRNAs and 2448 DEmRNAs, were normalized by voom function using the Limma package (Figure 2). TCGA-G-A2C8-11, an obvious outlier sample based on gene expression, was excluded (Supplementary Figure 1A). The clinical trait heatmap and sample dendrogram divided the selected samples into different clusters and provided a distribution map of clinical trait data (Supplementary Figure 1B), including age at initial pathologic diagnosis (a), pathologic_M (b), pathologic_N (c), pathologic_T (d), tumor stage (e), additional pharmaceutical therapy (f), radiation therapy (g), vital status (h), days to new tumor event after initial treatment (i), and days to death (j) (Supplementary Table 4 and Supplementary Figure 1B).

We used the WGCNA algorithm to construct a co-expression network and modules for the 1210 samples. The Pearson’s correlation matrix of the genes was converted into a strengthening adjacency matrix by power β = 4 based on a scale-free topology with R^2^ = 0.97 (Figure 2A). All of the selected genes were clustered using a topological overlap matrix (TOM)-based dissimilarity measure based on the Dynamic Tree Cut algorithm to divide the tree into eight modules (Figure 2B) labeled with different colors. The number of genes in each module is shown in Table 1. Next, Pearson’s correlation coefficient was used to analyze the interaction of these co-expression modules. Hierarchical clustering of module eigengenes summarizing the modules was found in the clustering analysis. Branches of the dendrogram (the meta-modules) were grouped together based on the correlation of eigengenes (Figure 2C). Each module contained different gene clusters and was labeled by a different color in the heatmap plot of topological overlap; red represented a positive correlation, while blue represented negative correlation (Figure 2D).

**Table 1:** The number of genes in the co-expression modules.

**Module**	**Genes**
Turquoise	1296
Green	68
Blue	1071
Red	42
Yellow	130
Brown	453
Black	35
Grey	206

### Identification of key modules and hub genes related to BRCA prognosis

**Figure 3:**
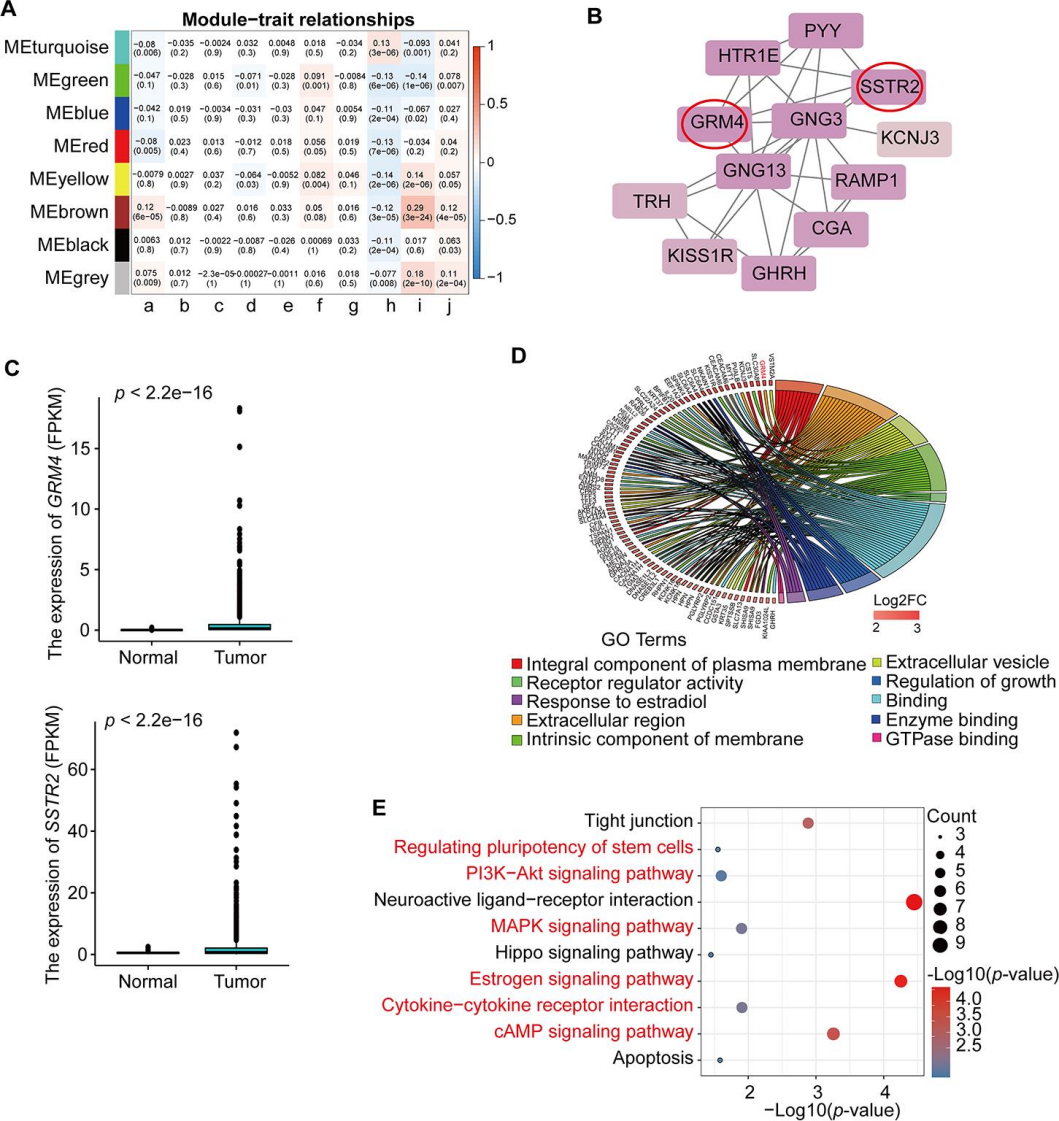
**Identification and analysis of key module and hub genes.** (**A**) Analysis of module-trait relationships of BRCA based on TCGA data; a. age at initial pathologic diagnosis, b. pathologic_M, c. pathologic_N, d. pathologic_T, e. tumor stage I, f. additional pharmaceutical therapy, g. radiation therapy, h. vital status, i. days to new tumor event after initial treatment, j. days to death. TNM = tumor, node, metastasis (classification). (**B**) PPI analysis and identification of hub genes involved in the co-expression Brown module using STRING database and MCODE plug-in in Cytoscape. The genes in the red circle are the hub genes. (**C**) Expression of *GRM4* and *SSTR2* in BRCA from TCGA database. (**D**) GO enrichment in the co-expression Brown module. The red gene is the hub gene of PPI. (**E**) KEGG pathway enrichment in the co-expression Brown module. Red pathways are common with total DEmRNAs.

We summarized the gene co-expression by eigengenes and calculated the correlation of each eigengene with clinical traits, such as age at initial pathologic diagnosis (a), pathologic_M (b), pathologic_N (c), pathologic_T (d), tumor stage I (e), additional pharmaceutical therapy (f), radiation therapy (g), vital status (h), days to new tumor event after initial treatment (i), and days to death (j) (Figure 3A). The module-trait relationship plot showed that the co-expression Brown module was most significantly positively associated with days to new tumor event after initial treatment (i) (R=0.29, p=3e-24) and days to death (R = 0.12, *p* = 6e-05) and negatively associated with vital status (h) (R = ‒0.12, *p* = 3e-05). The co-expression Yellow module was significantly positively associated with days to new tumor event after initial treatment (i) (R=0.14, *p*= 2e-06) and negatively associated with vital status (h) (R=‒0.14, *p*=2e-06); the co-expression Turquoise module was significantly positively associated with vital status (h) (R=0.13, *p*=3e-06) and negatively associated with new tumor event after initial treatment (i) (R=‒0.093, *p*=0.001). Thus, the co-expression Brown module (Supplementary Table 5) was the key module for BRCA prognosis and was used for further analysis.

As shown in Figure 3B, we constructed a PPI network using the STRING database and the Molecular Complex Detection (MCODE) plug-in in Cytoscape (MCODE score >10) for the 453 genes from the co-expression Brown module. The results demonstrated that *GRM4* and *SSTR2* were potential hub genes that interact with other genes (Figure 3B). In addition, the expression levels of these two hub genes were higher in BRCA than in normal samples (Figure 3C). For further analysis, we used the KOBAS online database to analyze the GO and KEGG pathway enrichment of the 453 genes in the co-expression Brown module. The significant enrichment function and pathways (*p*<0.05) are shown in Figure 3D, 3E. GO data revealed that the genes were enriched in integral components of the plasma membrane, extracellular region, extracellular vesicle, intrinsic component of membrane, receptor regulator activity, regulation of growth, enzyme binding, response to estradiol, and GTPase binding molecular function (Figure 3D, Supplementary Table 6). In addition, 10 distinct KEGG signaling pathways possibly related to BRCA were identified (Figure 3E, Supplementary Table 6), such as signaling pathways regulating the pluripotency of stem cells, PI3K-Akt signaling pathway, MAPK signaling pathway, estrogen signaling pathway, cytokine-cytokine receptor interaction, and the cAMP signaling pathway, which are common pathways in total differential mRNAs KEGG pathway enrichment (Table 2). The hub genes *GRM4* and *SSTR2* were enriched in the neuroactive ligand-receptor interaction and cAMP signaling pathway, respectively. All the above pathways play a vital role in tumorigenesis.

**Table 2:** The common KEGG pathways enriched in the co-expression brown module and DEmRNA.

**KEGG pathways**	***p*-value**	**Gene list**
Signaling pathways regulating pluripotency of stem cells	2.802E-02	BMPR1B; HOXB1; FGFR3
PI3K-Akt signaling pathway	2.501E-02	CREB3L1; GNG13; GNG3; FGFR3; EIF4E1B
Hippo signaling pathway	3.549E-02	AMH; BMPR1B; PARD6B
MAPK signaling pathway	1.253E-02	CACNA1H; CACNG1; CACNG4; FGFR3; MAPK8IP2
Estrogen signaling pathway	5.579E-05	KCNJ3; KRT31; KRT35; TF1; KRT37; CREB3L1
Cytokine-cytokine receptor interaction	1.237E-02	AMH; BMPR1B; GDF15; TNFRSF18; IL20
cAMP signaling pathway	5.524E-04	AMH; GRIA2; SSTR2; CREB3L1; TNNI3; HTR1E

### Construction of lncRNA-miRNA-mRNA ceRNA and lncRNA-RBP-mRNA networks

**Figure 4:**
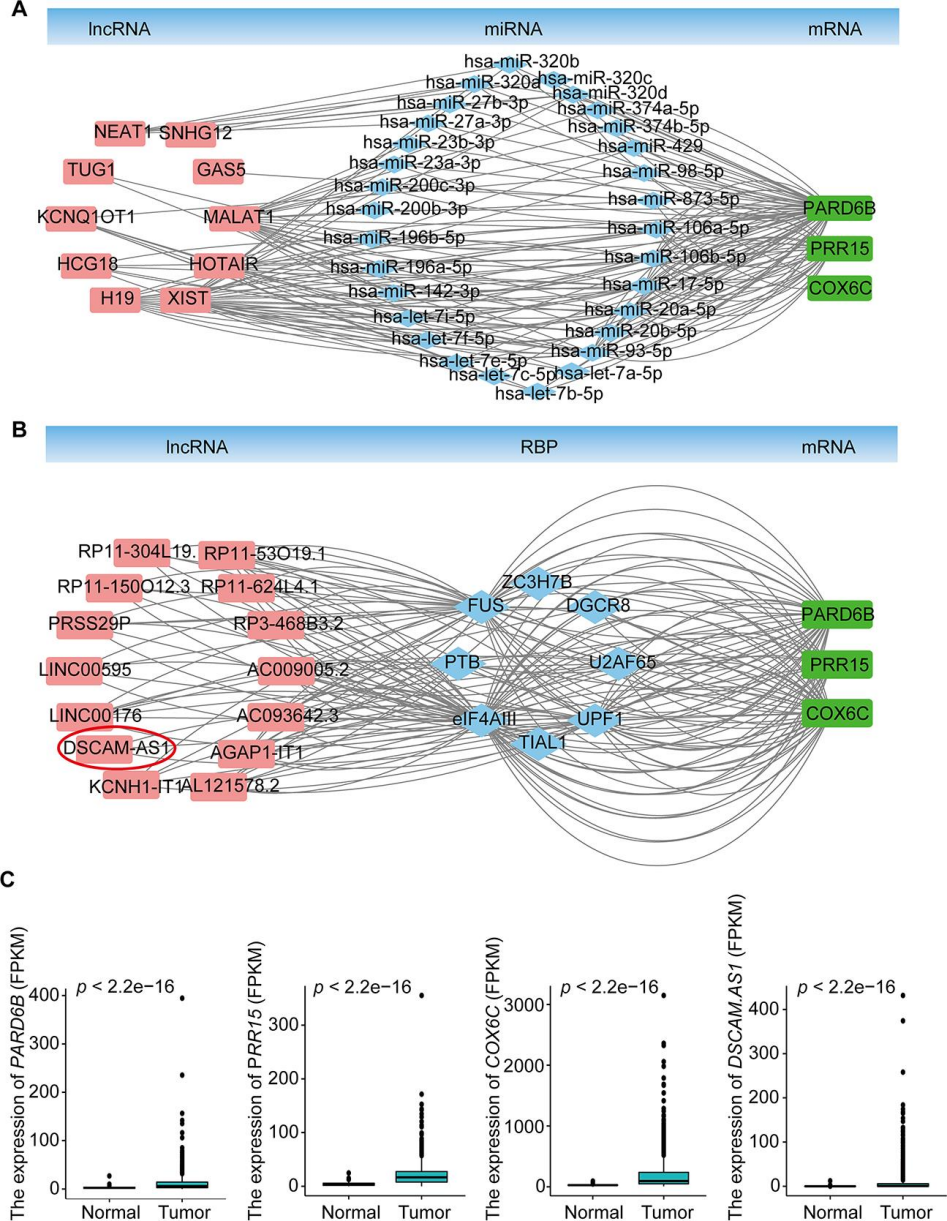
**lncRNA-miRNA-mRNA ceRNA and lncRNA-RBP-mRNA networks.** (**A**) lncRNA-miRNA-mRNA ceRNA network based on the co-expression Brown module. (**B**) lncRNA-RBP-mRNA network based on the co-expression Brown module. (**C**) Expression of *PARD6B, PRR15,* *COX6C,* and *DSCAM-AS1*.

To explore the molecular mechanism of BRCA-related lncRNA, lncRNA-miRNA-mRNA and lncRNA-RBP-mRNA networks were constructed from the starBase database, according to the DElncRNAs and DEmRNAs from the co-expression Brown module. The lncRNA-miRNA-mRNA ceRNA network consisted of 15 DElncRNAs, 57 DEmiRNAs, and 11 DEmRNAs (Supplementary Figure 2A, Supplementary Table 7). The lncRNA-RBP-mRNA network consisted of 45 DElncRNAs, 158 DEmRNAs, and 33 RBPs (Supplementary Figure 2B, Supplementary Table 7). We selected three genes (*PARD6B*, *PRR15*, and *COX6C*) that were significantly upregulated in BRCA (Figure 4C), as hub genes, according to the degree of lncRNA, miRNA, or RBP and previous studies of these genes in the two networks. These three genes were connected with 10 DElncRNAs (*MALAT1*, *XIST*, *NEAT1*, *TUG1*, *HCG18*, *KCNQ1OT1*, *H19*, *GAS5*, *SNHG12*, and *HOTAIR*), 30 DEmiRNAs in the lncRNA-miRNA-mRNA ceRNA network and 14 DElncRNAs (*AC009005.2*, *AC093642.3*, *AGAP1-IT1*, *AL121578.*2, *DSCAM-AS1*, *KCNH1-IT1*, *LINC00176*, *LINC00595*, *PRSS29P*, *RP11-150O12.3*, *RP11-304L19.12*, *RP11-53O19.1*, *RP11-624L4.1*, and *RP3-468B3.2*), and 8 RBPs (U2AF65, UPF1, TIAL1, eIF4AIII, PTB, FUS, ZC3H7B, and DGCR8) in the lncRNA-RBP-mRNA network (Figure 4A, 4B, Supplementary Table 8). Previous studies have found that *DSCAM-AS1* is highly specific to luminal breast cancer and is directly regulated by estrogen receptor α (ERα), playing vital roles in tumor proliferation, invasion, and tamoxifen resistance [22–24]. Our results indicated that *DSCAM-AS1* was highly expressed in the BRCA samples (Figure 4C) and bound with RBP OPF1 and mRNA *PARD6B*, suggesting that *DSCAM-AS1* may work with OPF1 and *PARD6B* to promote BRCA progression.

### Validation of the expression of the selected hub genes

**Figure 5:**
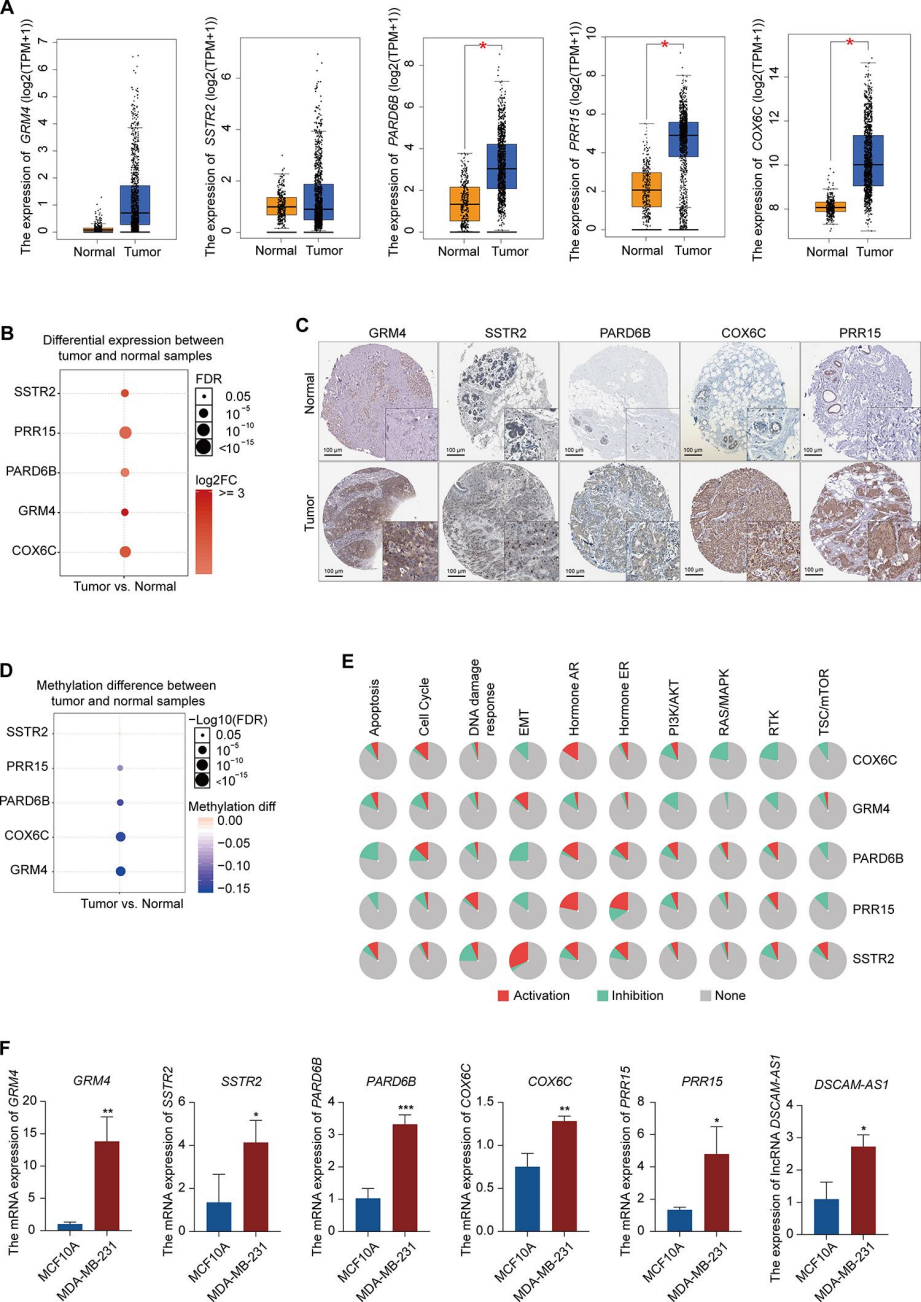
**Expression pattern validation of hub genes and signaling pathways in BRCA.** (**A**) Expression pattern of *GRM4*, *SSTR2*, *PARD6B*, *COX6C*, and *PRR15* in BRCA and normal samples from the GEPIA database. (**B**) Expression of *GRM4*, *SSTR2*, *PARD6B*, *COX6C*, and *PRR15* in BRCA and normal samples from the GSCALite database. (**C**) IHC of the GRM4 (GRM4 normal sample from 2104; GRM4 BRCA sample from 2160), SSTR2 (SSTR2 normal sample from 3286; SSTR2 BRCA sample from 2091), PARD6B (PARD6B normal sample from 2042; PARD6B BRCA sample from 1874), COX6C (COX6C normal sample from 2773; COX6C BRCA sample from 1775), and PRR15 (PRR15 normal sample from 2773; PRR15 BRCA sample from 2428) in BRCA and normal samples from the HPA database. (**D**) Difference in the methylation of *GRM4*, *SSTR2*, *PARD6B*, *COX6C*, and *PRR15* between BRCA and normal samples from the GSCALite database. (**E**) Difference in the signaling of pathways associated with *GRM4*, *SSTR2*, *PARD6B*, *COX6C*, and *PRR15* between BRCA and normal samples from the GSCALite database. (**F**) Expression of *GRM4*, *SSTR2*, *PARD6B*, *COX6C*, *PRR15*, and lncRNA *DSCAM-AS1* in MCF10A (normal breast epithelial cell line) and MDA-MB-231 (breast cancer cell line) using qRT-PCR.

To confirm the reliability of the five differentially expressed genes (*GRM4*, *SSTR2*, *PARD6B*, *COX6C*, and *PRR15*) from PPI, lncRNA-miRNA-mRNA ceRNA, and lncRNA-RBP-mRNA network, we verified the expression patterns of these genes in multiple databases. The mRNA expression levels of *PARD6B*, *COX6C*, and *PRR15* were significantly higher in BRCA than in normal samples, according to the Gene Expression Profiling Interactive Analysis database. The mRNA expression levels of *GRM4* and *SSTR2* was increased in BRCA, although not significantly (Figure 5A). All of these hub genes were significantly increased in BRCA, according to the GSCALite database (Figure 5B). In addition, the protein expression levels of these five genes were significantly higher in tumor samples than in normal samples, according to the Human Protein Atlas (HPA) database (Figure 5C). We also analyzed the methylation of these genes in the GSCALite database and found that *GRM4*, *PARD6B*, *COX6C*, and *PRR15* were hypomethylated in BRCA (Figure 5D) which may be related to the high expression of these genes in BRCA. Finally, *GRM4* and *SSTR2* were found to be activated in the EMT pathway in BRCA, while *PARD6B* and *PRR15* were inhibited. *SSTR2*, *PARD6B*, and *PRR15* were activated in the ER pathway. *COX6C* and *GRM4* were inhibited in the PI3K/AKT, RAS/MAPK, and RTK pathways (Figure 5E). These pathways play vital roles in oncogenesis, suggesting that these hub genes may participate in BRCA progression. Furthermore, these five hub mRNAs and lncRNA *DSCAM-AS1* expression in MCF10A (normal breast epithelial cell line) and MDA-MB-231 (TNBC cell line) were measured using qRT-PCR. Expression levels of *GRM4*, *SSTR2*, *PARD6B*, *COX6C*, *PRR15*, and *DSCAM-AS1* were significantly higher in MDA-MB-231 than in MCF10A (Figure 5F), which were consistent with the expression pattern of these six genes in multiple databases.

## DISCUSSION

BRCA is the most commonly diagnosed malignancy among women worldwide. Although the number of breast cancer survivors is rising thanks to increasingly early diagnoses and improved therapy treatments [25]. However, the number of women who experience recurrence associated with an unexpected prognosis after the primary tumor is diagnosed, such as distant metastases and poor quality of life, is also increasing [26–28]. Thus, the identification of noninvasive biomarkers with high sensitivity and specificity for use in breast cancer detection at an early stage and in monitoring the response to therapy is vital to improve prognosis. LncRNA, traditionally considered transcriptional noise, is now known to be involved in genome packaging, chromatin organization, dosage compensation, genomic imprinting, and gene regulation [29]. Increasing evidence has demonstrated that lncRNAs are critical components during cancer initiation, development, and progression [30]. Specific lncRNAs are now likely to be translated into clinical applications for diagnosis, prognosis, and prediction of treatment response [31].

WGCNA is the most widely used co-expression network technique and has been used in many applications, for example, in the genetic analysis of cancer, genome analysis in mice and yeast, and the analysis of brain MRI data [32]. WGCNA is notably useful for identification of the modules of co-expressed genes that are correlated with clinical traits and consequently biological tumor behavior. Huang et al. identified hub gene CDC45 as a putative novel therapeutic target in NSCLC through WGCNA analysis. WGCNA was also used to determine hub genes, lncRNAs and miRNAs correlated with BRCA progression and prognosis in previous studies. For example, Yao et al. have constructed 23 modules using weighted gene co-expression network analysis and identified 5 lncRNAs associated with BRCA progression from Green module and Blue module which were positively correlated with tumor samples [33]. Liu et al. have identified breast cancer-related preserved modules among 4 individual GSE datasets using weighted gene co-expression network analysis and selected eight lncRNAs as prognostic biomarkers using univariate Cox regression analysis in combination with LASSO analysis [34]. Moreover, WGCNA can also be used for selecting hub genes and prognostic biomarkers associated with different subtypes of breast cancer. For example, Adhami M. et al have identified 2 or 3 miRNAs as novel biomarkers for each subtype of breast cancer using WGCNA co-expression analysis [35].

Although many efforts have been made to identify prognostic biomarkers in BRCA using WGCNA, clinical prognostic traits haven’t been taken into consideration. In our studies, we first screened differential expressed lncRNAs and mRNAs between normal samples and breast cancer samples and then carried out WGCNA analysis using 10 clinical traits, including days to new tumor event after initial treatment and days to death. We identified 8 modules and found that the co-expression Brown module was the one most significantly correlated with days to new tumor events after initial treatment and days to death. We selected Brown module as the key module of prognosis. DEmRNAs and DElncRNAs in Brown module were used for selecting hub genes associated with breast cancer prognosis and construct ceRNA network and lncRNA-RBP-mRNA network.

Our results indicated that *GRM4* and *SSTR2* were hub genes for BRCA prognosis using PPI network analysis in BRCA. GRM4, a member of the G protein-coupled receptor family, can directly couple with ion channels through G protein mediation to increase cell excitability and activate the second messenger and downstream signal transduction system [36]. Previous studies have demonstrated that GRM4 may have different functions in various cancers. In renal cell carcinoma, GRM4 was previously found to be highly expressed and correlated with poor prognosis compared with that in normal samples [37]. However, GRM4 can inhibit the proliferation and DNA synthesis of various medulloblastoma cell lines by inhibiting the cAMP and IP3K pathways [38]. SSTR2 is also a G-protein coupled plasma membrane receptor [39]. Study has shown that SSTR2 can inhibit cell proliferation by upregulating p21 and p16 or increasing caspase-3 and decreasing PARP expression in human pancreatic and lung cancer cell lines [40]. Moreover, early studies have shown that SSTR2 is the most widely expressed SSTR subtype in breast cancer [41, 42]. MCF7 cells with high levels of SSTR2 expression display a diminished rate of cell proliferation by MAPK, PI3K/AKT, and phosphotyrosine phosphatase pathways [43, 44]. Given that *GRM4* and *SSTR2* were significantly higher in the BRCA samples than in the normal samples, in addition to the enrichment of GTPase binding molecular function, cAMP, PI3K-Akt, and MAPK signaling pathways in the co-expression Brown module, our results suggested that *GRM4* and *SSTR2* play a vital role in BRCA metastasis and prognosis by regulating these pathways and may thus be used as therapeutic targets for BRCA patients.

Furthermore, the lncRNA-miRNA-mRNA ceRNA and lncRNA-RBP-mRNA networks indicated that several lncRNAs may participate in BRCA progression and diagnosis, such as *H19* and *MALAT1*. Several studies have found that H19 participates in the carcinogenic process [45, 46]. Moreover, the *H19/let-7/Lin28* ceRNA network is capable of inhibiting the epithelial-mesenchymal transition by downregulating autophagy in BRCA [47]. A previous study reported that *H19* can competitively bind *miR-93-5p* to upregulate STAT3 and promote proliferation, migration, and invasion in breast cancer [48]. Other studies have suggested that *H19* acts as an miR-675-5p and *miR-340-3p* sponge to induce breast cancer cell apoptosis and promote epithelial-mesenchymal transition in paclitaxel-resistant breast cancer cells, respectively [49, 50]. *MALAT1* is a metastasis-suppressing lncRNA that is highly expressed in breast cancer tissues and is associated with disease progression [51]. In addition, *MALAT1* can also bind with multiple miRNAs, such as *miR-1*, *miR-129-5p*, and *miR-145*, to promote BRCA [52–54]. Similar to previous studies, our ceRNA network results indicated that *H19* and *MALAT1* may work with different miRNAs to promote BRCA. However, the mechanisms by which *MALAT1* and *H19* act with these miRNAs to promote the progression of BRCA will need to be analyzed further.

*PARD6B*, *PRR15*, and *COX6C* were selected as hub genes in the lncRNA-miRNA-mRNA ceRNA and lncRNA-RBP-mRNA networks. Our results indicated that these three genes may work with multiple lncRNAs, miRNAs, and RBPs to regulate BRCA progression. According to previous reports, these three genes play an important role in different cancers. PARD6B is an essential component in epithelial cell tight junction (TJ) formation and the maintenance of apico-basal polarity. PARD6B overexpression promotes the activation of MAPK and cell proliferation in breast cancer [55]. PARD6B inhibition in MCF7 cells resulted in the loss of tight junctions [56]. COX6C, a mitochondrial inner membrane protein, was highly prevalent in the plasma of melanoma patients as well as in ovarian and breast cancer patients [57]. These studies indicated that *PARD6B* and *COX6C* may participate in BRCA progression.

In conclusion, our study used RNA-seq expression data from the TCGA database and compared DElncRNAs and DEmRNAs between normal and BRCA samples. WGCNA was used to construct the free-scale network, which was combined with phenotype information to further identify the co-expression Brown module. This module was found to be significantly associated with BRCA prognosis. PPI network analysis and gene function enrichment were performed to identify the hub genes (*GRM4* and *SSTR2*) and key pathways in the co-expression Brown module. Furthermore, lncRNA-miRNA-mRNA ceRNA and lncRNA-RBP-mRNA networks were established to demonstrate that *PARD6B*, *PRR15*, *COX6C*, and *DSCAM-AS1* were activated in BRCA. Meanwhile, these genes were verified in multiple databases and using qRT-PCR, which were significantly highly expressed in BRCA. However, in this work, we constructed a synthetic network based on multiple genes, with the impact of single genes on the BRCA mechanism being unclear. Thus, further experimental data will be needed to support this investigation and confirm the detailed molecular mechanisms in BRCA progression.

## MATERIALS AND METHODS

### TCGA data of BRCA patients and data preprocessing

**Figure 6:**
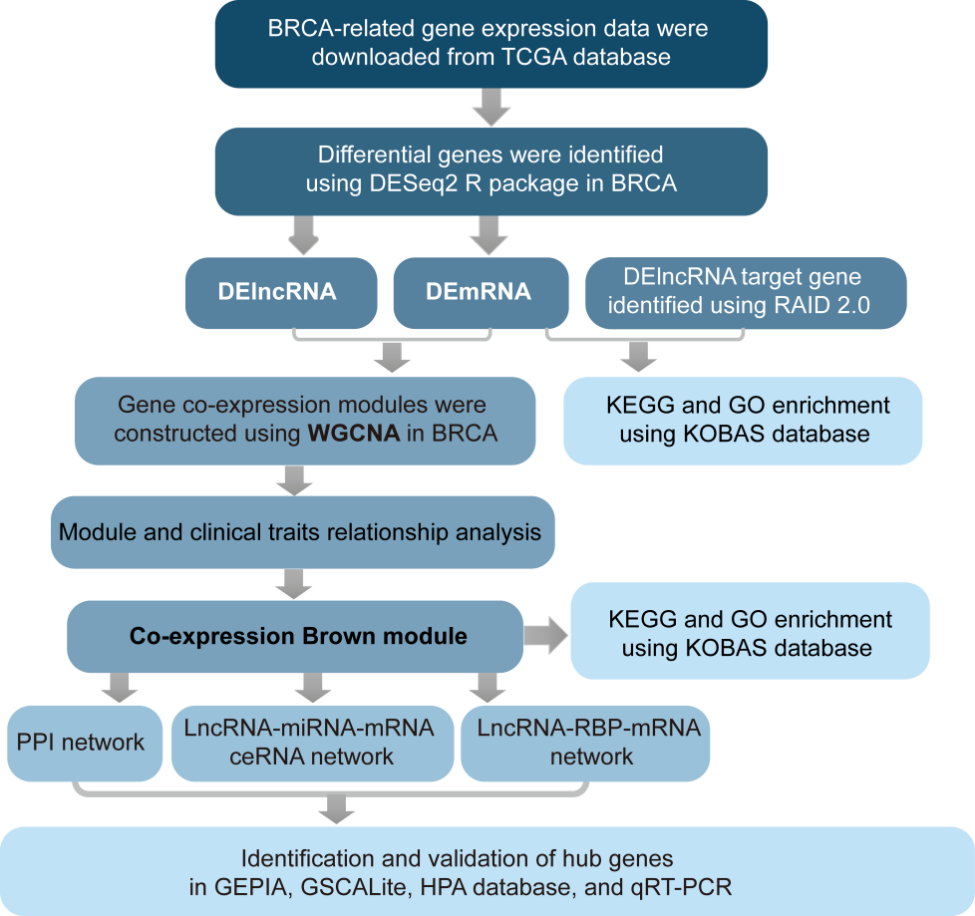
**Flow chart of analysis.**

The workflow of data analysis is shown in Figure 6. BRCA-related RNA-seq data (1217 samples, including 1104 tumors and 113 normal controls), prognostic data (1104 samples), and related clinical trait data (1266 samples) were downloaded from the TCGA database (https://portal.gdc.cancer.gov/projects/TCGA-BRCA), as shown in Table 3. Gene expression levels of TCGA-A7-A0DB-01, TCGA-A7-A13E-01, TCGA-A7-A13D-01, TCGA-A7-A0DC-01, TCGA-A7-A26J-0, and TCGA-A7-A26E-01 were set as replicated sample means because of their replication. Ultimately, 1098 tumor samples were used for further analysis. A total of 3301 differential genes were identified between the tumor and normal samples using the DESeq2 R package, at thresholds of |log2FC| > 2 and adj-*p* < 1e-3. Differentially expressed lncRNAs (DElncRNAs) and mRNAs (DEmRNAs) were used for further analysis.

**Table 3:** Information of BRCA patients clinical traits.

**Clinical traits of BRCA patients**	**Case, n (%)**
**Total**	1266
**Age at initial pathologic diagnosis**	58.22+13.38
**Days to new tumor event after initial treatment**	977.6531+780.17
**Pathologic T**	
**T1**	323 (25.5)
**T2**	736 (58.1)
**T3**	155 (12.2)
**T4**	49 (3.9)
**TX**	3 (0.2)
**Pathologic N**	
**N0**	585 (46.2)
**N1**	434 (34.3)
**N2**	135 (10.7)
**N3**	89 (7.0)
**NX**	23 (1.8)
**Pathologic M**	
**M0**	1061 (83.8)
**M1**	24 (1.9)
**MX**	181 (14.3)
**Radiation therapy**	
**Yes**	615 (48.6)
**No**	w484 (38.2)
**Unknown**	167 (13.2)
**Additional pharmaceutical therapy**	
**Yes**	26 (2.0)
**No**	20 (1.6)
**Unknown**	1220 (96.4)
**Days to death**	1568.91+ 1228.10
**Vital status**
**Alive**	1054 (83.3)
**Dead**	212 (16.7)
**Tumor stage**
**Stage I**	212 (16.7)
**Stage II**	720 (56.8)
**Stage III**	287 (22.7)
**Stage IV**	22 (1.7)
**Stage X**	13 (1.0)
**Unknown**	12 (1.0)

### Gene function enrichment of DElncRNA target genes and mRNAs

Target genes of DElncRNA were obtained using the RAID 2.0 database (https://www.rna-society.org/raid/) and overlapped with DEmRNAs. Functional and pathway enrichment of DEmRNAs was performed using the KOBAS online database (http://kobas.cbi.pku.edu.cn/kobas3). Significantly enriched functions and pathways were visualized by R package ggplot2 with RStudio (Version 3.6.3).

### WGCNA of DElncRNAs and DEmRNAs

WGCNA is a systematic biological method used to construct a scale-free network based on gene expression profiles. To construct this system, a similarity matrix that calculates the absolute value of the Pearson’s correlation coefficient between two genes was constructed using expression data. Then, the similarity matrix was converted into adjacency matrix a_ij_, where the β value was the soft-threshold (power value) to enhance strong connections and disregard weak correlations between genes in the adjacency matrix. Next, the adjacency matrix was converted into a TOM to describe the association strength between the genes. TOM was used as an input for the hierarchical clustering analysis of genes, and the DynamicTreeCut algorithm was applied to identify network modules. The most representative genes, module eigengenes (MEs), were the first principal components, representing the overall level of gene expression in individual modules. Module membership (MM) was measured using Pearson’s correlation coefficient of the expression profile of one gene in all samples and one ME. Lastly, the gene significance (GS) was used to evaluate the gene with other biological information. The higher the value of GS, the more prognostic value it holds for the patient. Thus, the expression profile of DElncRNAs and DEmRNAs was used to construct a free-scale network and identify significant modules related to clinical traits to analyze differential genes in these modules.

### Construction of the PPI, lncRNA-miRNA-mRNA ceRNA, and lncRNA-RBP-mRNA network of the Brown module

The online STRING database (https://string-db.org/) was used to build the PPI network. The network graph was visualized and analyzed using Cytoscape v3.6.0. Then, the hub mRNAs were selected with a cutoff score of 10 using the MCODE plug-in. The lncRNA-miRNA-mRNA ceRNA and lncRNA-RBP-mRNA networks in the co-expression Brown module were constructed based on starBase (http://starbase.sysu.edu.cn/) and visualized in Cytoscape.

### Verification of the expression pattern and identification of pathway signaling of hub genes

The mRNA expression patterns of the hub genes in BRCA and normal samples were verified using the GEPIA (http://gepia.cancer-pku.cn/) and GSCALite database (http://bioinfo.life.hust.edu.cn/web/GSCALite/), a Web server for Gene Set Cancer Analysis. Protein expression of the hub genes between BRCA and normal tissues was determined using immunohistochemistry (IHC) from the HPA (https://www.proteinatlas.org/). HPA is a valuable database that provides a large amount of transcriptomics and proteomics data for specific human tissues and cells. The pathway signaling of hub genes was analyzed in the GSCALite database.

### Cell culture

The MCF10A and MDA-MB-231 cells here were all originally purchased from American Type Culture Collection (Manassas, VA, USA) and were used to verify the expression of hub genes in this study. MCF10A cells were cultured in the base medium for this cell line (MEBM) supplemented with 100 ng/ml cholera toxin. were cultured in Dulbecco’s modified Eagle’s medium (DMEM) supplemented with 10% fetal bovine serum and 1% antibiotics. Cells were maintained in a humidified incubator equilibrated with 5% CO2 at 37° C.

### Verification of the hub genes using qRT-PCR

Total cellular RNAs were extracted from MCF10A and MDA-MB-231 by using TRIzol (Invitrogen). cDNA was prepared using MMLV Reverse Transcriptase (Roche) and amplificated using 2 × PCR SYBR Green Mix buffer in a 15-μL reaction. The PCR process run 40 cycles of 95° C for 15s and 60° C for 1 min in ABI PRISM 7500 sequence-detection system (Applied Biosystems, Foster City, CA, USA). The results were shown by using the comparative Ct method (2-ΔΔCt) with β-actin as an internal control. The primers used were supplied in Supplementary Table 9.

### Statistical analysis

RStudio software 3.4.3 and SPSS were used to analyze BRCA sample data and qRT-PCR results, respectively. For comparisons between two groups, Student’s t test of variance was performed. *P*< 0.05 was used as statistically significant. All data were visualized using the GraphPad Prism 8 and RStudio (Version 3.6.3) software.

## Supplementary Material

Supplementary Figures

Supplementary Table 1

Supplementary Table 2

Supplementary Table 3

Supplementary Table 4

Supplementary Table 5

Supplementary Table 6

Supplementary Table 7

Supplementary Table 8

Supplementary Table 9
