# Supplementary figures and images for: Identification of key modules and genes associated with breast cancer prognosis using WGCNA and ceRNA network analysis
Source: Aging (Albany NY). 2020 Dec 9;13(2):2519–38. doi: 10.18632/aging.202285 (PMC7880379; doi:10.18632/aging.202285)

[www.aging-us.com](http://www.aging-us.com)

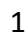

Supplement: Supplementary Figures [file aging-13-202285-s001.pdf]
