# Supplementary material for: Identification of key modules and genes associated with breast cancer prognosis using WGCNA and ceRNA network analysis
Source: Aging (Albany NY). 2020 Dec 9;13(2):2519–38. doi: 10.18632/aging.202285 (PMC7880379; doi:10.18632/aging.202285)
Supplement: Supplementary Table 9 [file aging-13-202285-s010.pdf]

Supplementary Table 9. The primers of hub genes measured using qRT-PCR.

Gene	Forward primer (5' → 3')	Reverse primer(5' → 3')
GRM4	GTGTCATCGGTGCTTCAGGG	CGCGGGAGAAGAAGTCGTAG
SSTR2	GCTGTGCCAACCCTATCCTA	CTTACTGTCACTCCGCTCCC
PARD6B	ATGGAGGTGAAGAGCAAGTT	ATGCGTTTCTGGGAGAATAT
COX6C	TATGGCTGTAGCATTCGTGC	GCGTATGCCTTCTTTCTTTG
PRR15	AAAGCAAGGAAGCCGCAGTG	AGATTGGGGTGCTGGTTCTC
DSCAM-AS	TCCTGGAAGAGGTGGGTTAT	TGTTGTGGTTTTGAGATGGG
β-actin	CTCGCCTTTGCCGATCC	TCTCCATGTCGTCCCAGTTG
